# Development and Internal Validation of a Predictive Nomogram for Assessing Rhabdomyolysis Risk After Wasp Stings: A Multicenter Study in Sichuan, China

**DOI:** 10.1155/emmi/8817533

**Published:** 2026-07-29

**Authors:** Xiaoyan Xian, Guoqiang Chen, Jianping Hu, Yuanjun Zhang, Mengqin Li, Jing Chen, Jiao Li, Shuyun Xu

**Affiliations:** ^1^ Department of Emergency Medicine, Laboratory of Emergency Medicine, West China Hospital, and Disaster Medical Center, Sichuan University, Chengdu 610041, Sichuan, China, scu.edu.cn; ^2^ Department of Emergency Medicine, Guangyuan Central Hospital, Guangyuan 628000, Sichuan, China, vcu.edu; ^3^ Department of Emergency Medicine, Suining Central Hospital, Suining 629000, Sichuan, China, sns120.cn; ^4^ Department of Emergency Medicine, Ziyang Central Hospital, Ziyang 641300, Sichuan, China, scu.edu.cn; ^5^ Department of Emergency Medicine, Affiliated Hospital of North Sichuan Medical College, Nanchong 638000, Sichuan, China, hospital-nsmc.com.cn; ^6^ Department of Emergency, Shangjin Nanfu Hospital, West China Hospital, Sichuan University, Chengdu 610044, Sichuan, China, scu.edu.cn

**Keywords:** multicenter study, nomogram, rhabdomyolysis, wasp sting

## Abstract

Wasp venom–induced rhabdomyolysis (RM) is a serious complication associated with poor clinical prognosis. However, predictive models for RM after wasp stings remain limited. This study aimed to develop and internally validate a clinical prediction model for RM in patients with wasp stings. This multicenter retrospective cohort study included 607 patients admitted to five tertiary hospitals in Sichuan Province for wasp stings between February 2015 and December 2020. Least absolute shrinkage and selection operator (LASSO) regression and multivariate logistic regression were used to identify independent predictors of RM. A nomogram incorporating nine predictors was constructed. Model performance was assessed by internal validation and decision curve analysis (DCA). Among the 607 patients, 178 (29.3%) developed RM. Nine predictors were included in the final model: age, sting species, number of stings, tea‐colored urine, white blood cell count (WBC), lactate dehydrogenase( LDH), total bilirubin (TBIL), activated partial thromboplastin time (APTT), and month of injury. The nomogram showed excellent discrimination, with an area under the receiver operating characteristic curve (AUC) of 0.949 (95% CI, 0.9319–0.9655) and a concordance index (C‐index) of 0.948. DCA suggested good potential clinical utility of the model. We developed a nomogram for early prediction of RM risk after wasp stings using readily available clinical variables. This model may help identify high‐risk patients at an early stage and support timely clinical management.

## 1. Introduction

The Hymenoptera order encompasses a vast array of insect species. Bees and wasps belong to hymenoptera [1]. Hymenoptera stings are a common cause of insect related injury, especially in rural areas. The cases happened suddenly. In most cases, stings cause only localized reactions. However, in severe cases, it may cause anaphylactic shock, rhabdomyolysis (RM), multiple organ dysfunction, acute kidney and hepatic injury, blood coagulation issues, and potentially death [2–4].

RM presents with a clinical spectrum ranging from isolated elevation of creatine phosphokinase (CK) levels to life‐threatening conditions characterized by severe electrolyte disturbances and acute kidney injury (AKI). Approximately 10%–50% of patients develop AKI, which results from multiple pathogenic mechanisms, including hypovolemia‐induced renal ischemia, direct tubular toxicity of myoglobin, renal vasoconstriction, and intratubular cast formation [5]. Xie et al. reported that among 311 patients with bee stings, 167 had elevated CK levels, 75 were diagnosed with RM, and 58 developed AKI. The incidence of AKI in patients with bee venom–induced RM (75.9%) was significantly higher than that in the overall bee sting population (21%) [3].

Previous studies have mainly focused on the occurrence and mechanisms of AKI postwasp stings [6, 7], with less attention paid to its precursor, RM. However, RM is an important component of the systemic toxic reaction induced by wasp venom and one of the major triggers of AKI. Although RM is linked to AKI, not all patients with RM develop AKI. This observation underscores the notion that RM possesses its own distinct clinical significance and value in risk identification. Therefore, the early identification of wasp sting–induced RM is crucial for improving clinical outcomes. In clinical practice, however, early identification of patients at high risk for RM remains challenging, and practical tools for risk stratification are lacking. In addition, evidence derived from single‐center studies may have limited generalizability. Therefore, using multicenter clinical data, the present study aimed to develop and validate a prediction model for RM after wasp stings and to identify relevant independent predictors.

## 2. Materials and Methods

### 2.1. Study Design and Participants

**FIGURE 1:**
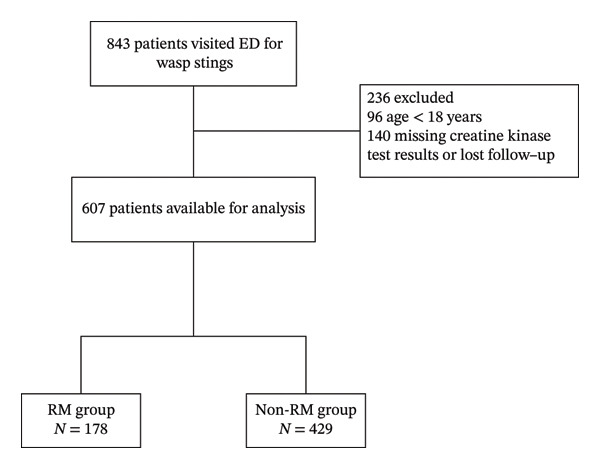
Study flowchart showing patient selection for analysis of wasp sting cases. ED: emergency department.

This was a retrospective, multicenter cohort study. Patients diagnosed with wasp stings between February 2015 and December 2020 were recruited from five clinical centers: West China Hospital of Sichuan University, Guangyuan Central Hospital, Suining Central Hospital, Ziyang Central Hospital, and Affiliated Hospital of North Sichuan Medical College. Sichuan Province is located in southwestern China, with a subtropical climate and mountainous terrain where wasp stings are relatively common. The diagnosis of Wasp sting was based on clinical history and physical examination. A total of 843 patients with wasp sting were initially enrolled. A total of 607 patients were included in the analysis after excluding 96 individuals under 18 years of age and 140 with missing creatine kinase test results or lost follow‐up (Figure 1).

The study complied with the Declaration of Helsinki principles and obtained ethical approval from the Ethics Committee of West China Hospital, Sichuan University (No. 2014156). The requirement for informed consent was waived because of the retrospective design of the study and the use of de‐identified patient data. The study was reported in accordance with TRIPOD‐AI guidelines for prediction model research.

The inclusion criteria were a confirmed history of wasp or bee stings together with relevant clinical manifestations.

The exclusion criteria were as follows: (1) a prior diagnosis of myositis; (2) absence of key laboratory data such as creatine kinase, liver and renal function, and electrolyte levels; (3) engagement in intense physical activity before the wasp sting; (4) age < 18 years; and (5) presentation to the hospital more than 3 days after the sting.

### 2.2. Outcomes and Other Definitions

The primary outcome was RM. The diagnostic criteria for RM were defined as a serum creatine kinase level ≥ 5 times the upper limit of normal (ULN), i.e., 1000 IU/L, detected at any time after wasp or bee sting injury [8]. Secondary outcomes included AKI, multiple organ dysfunction syndrome (MODS), acute toxic myocarditis, mortality, mechanical ventilation requirement, and blood purification therapy. AKI was diagnosed using the KDIGO criteria [9], which involve a serum creatinine increase of ≥ 0.3 mg/dL (≥ 26.4 μmol/L) within 48 h, a rise to ≥ 1.5 times the baseline within 7 days, or urine output ≤ 0.5 mL/kg/h for at least 6 h. MODS was identified according to established standards [10], requiring dysfunction or failure of at least two organ systems within 24 h of a wasp sting. Organ failure was defined as a sequential organ failure assessment (SOFA) value > 2 points. Acute toxic myocarditis is defined by elevated troponin T levels.

Data were extracted from the electronic medical record system. Collected variables included demographics (age and gender), sting species, number of stings, time from stings to admission, vital signs on admission (systolic and diastolic blood pressure, heart rate, respiratory rate, body temperature), clinical manifestations (gross hematuria, anuria or oliguria, chest tightness and pain, dizziness and headache, respiratory and circulatory failure), laboratory parameters (white blood cell count [WBC] and neutrophil count, hemoglobin, platelet count, blood urea nitrogen, creatinine, creatine kinase, lactate dehydrogenase [LDH], aspartate transaminase, alanine aminotransferase, direct, indirect, and total bilirubin [TBIL], prothrombin time, activated partial thromboplastin time [APTT], fibrinogen), and major therapeutic measures (mechanical ventilation, hemopurification). Sting species was classified based on patient or family report together with clinical judgment rather than formal entomological identification. All predictors included in the model were obtained within 24 h after presentation. The assessment, diagnosis, and management of wasp stings follow the guidelines outlined in the Expert Consensus Statement on Standardized Diagnosis and Treatment of Bee Stings in Sichuan [11].

### 2.3. Statistical Analysis Statistical

Statistical analyses were conducted using R software (v4.1.1) and SPSS (v26.0). Continuous variables were presented as mean ± SD or as the median with interquartile range. Categorical variables were presented as counts and percentages. Independent‐group *t*‐tests were used to compare means of continuous variables for normally distributed data, while the Mann–Whitney test was applied for non‐normally distributed data. Proportions were compared using either the χ^2^ test or Fisher’s exact test. For variables with missing data below 5%, multiple imputation was applied. The least absolute shrinkage and selection operator (LASSO) method is applied for analyzing high‐dimensional data and identifying key predictive factors [12]. A prediction model was developed using logistic regression. A nomogram was developed using the rms package in R. Model performance was assessed in terms of discrimination, calibration, and clinical utility. Discrimination was evaluated using the area under the receiver operating characteristic curve (AUC). Calibration was assessed using calibration plots, the Hosmer–Lemeshow test, and the calibration intercept and calibration slope. Internal validation was performed by bootstrapping (1000 bootstrap replicates) to calculate the corrected C‐index. Calibration plots were created to evaluate prediction accuracy and the alignment between predicted and actual severity levels [13]. The clinical utility of the nomogram was assessed using decision curve analysis (DCA). Model predictive accuracy was assessed via the AUC. A two‐tailed *p* value less than 0.05 was considered to be statistically significant.

## 3. Results

### 3.1. Clinical Characteristics of Patients

This study involved 607 patients treated for bee stings at five tertiary hospitals—West China Hospital of Sichuan University, Suining Central Hospital, Ziyang Central Hospital, Affiliated Hospital of North Sichuan Medical College, and Guangyuan Central Hospital—between February 1, 2015, and December 30, 2020. Among these patients, 28 died. The mean age was 53.24 ± 15.41 years, and 346 patients (57.0%) were male. Anaphylaxis occurred in 11 patients (1.8%), while tea‐colored urine was observed in 107 patients (17.6%). Oliguria or anuria developed early in 54 patients (8.9%), and chest tightness or chest pain occurred in 52 patients (8.6%). Dizziness or headache was reported in 124 patients (20.4%), while 14 patients (2.3%) developed early respiratory failure, and 14 patients (2.3%) developed early circulatory failure. The RM group comprised 178 patients, whereas the non‐RM group included 429 patients. In the RM group, acute liver injury occurred in 98 cases (55.1%), AKI in 99 cases (55.6%), acute toxic myocarditis in 103 cases (57.9%), MODS in 116 cases (65.2%), mechanical ventilation in 23 cases (12.9%), blood purification therapy in 110 cases (61.8%), in‐hospital cardiac arrest in 8 cases (4.5%), and death in 23 cases (12.9%). In the non‐RM group, acute liver injury occurred in 18 cases (4.2%), AKI occurred in 64 cases (14.9%), acute toxic myocarditis in 20 cases (4.7%), MODS in 18 cases (4.2%), mechanical ventilation in 7 cases (1.6%), blood purification therapy in 28 cases (6.5%), in‐hospital cardiac arrest in 1 case (0.2%), and death in 5 cases (1.2%). Table 1 provides a summary of the clinical and demographic characteristics of our cohort.

**TABLE 1:** Clinical characteristics in the RM and non‐RM groups.

	RM group (*N* = 178)	Non‐RM group (*N* = 429)	Total (*N* = 607)	*p* value
Age, years	60.21 ± 14.72	50.35 ± 14.77	53.24 ± 15.41	< 0.001
Sex				
Male	96 (53.9%)	250 (58.3%)	346 (57.0%)	0.325
Female	82 (46.1%)	179 (41.7%)	261 (43.0%)	
Sting species				
Honeybee	15 (8.4%)	236 (55.0%)	251 (41.4%)	< 0.001
Wasp	163 (91.6%)	193 (45.0%)	356 (58.6%)	
Number of stings				
< 15	60 (33.7%)	371 (86.5%)	431 (71.0%)	< 0.001
15–30	30 (16.9%)	24 (5.6%)	54 (8.9%)	
> 30	88 (49.4%)	34 (7.9%)	122 (20.1%)	
Anaphylactic shock				
No	171 (96.1%)	353 (99.2%)	596 (98.2%)	0.018
Yes	7 (3.9%)	3 (0.8%)	11 (1.8%)	
Gross hematuria				
No	84 (47.2%)	416 (97.0%)	500 (82.4%)	< 0.001
Yes	94 (52.8%)	13 (3.0%)	107 (17.6%)	
Anuria or oliguria				
No	135 (75.8%)	418 (97.4%)	553 (91.1%)	< 0.001
Yes	43 (24.2%)	11 (2.6%)	54 (8.9%)	
Chest tightness and pain				
No	146 (82.0%)	409 (95.3%)	555 (91.4%)	< 0.001
Yes	32 (18.0%)	20 (4.7%)	52 (8.6%)	
Dizziness and headache				
No	112 (62.9%)	371 (86.5%)	483 (79.6%)	< 0.001
Yes	66 (37.1%)	58 (13.5%)	124 (20.4%)	
Respiratory failure				
No	166 (93.3%)	427 (99.5%)	593 (97.7%)	< 0.001
Yes	12 (6.7%)	2 (0.5%)	14 (2.3%)	
Circulatory failure				
No	168 (94.4%)	425 (99.1%)	593 (97.7%)	< 0.001
Yes	10 (5.6%)	4 (0.9%)	14 (2.3%)	
SBP, mmHg	136 ± 28.33	129.28 ± 19.99	131.38 ± 22.96	0.034
DBP, mmHg	81.64 ± 16.82	81.11 ± 13.28	81.27 ± 14.40	0.681
HR, bpm	81.77 ± 16.86	83.28 ± 14.19	82.84 ± 15.03	0.261
Na, mmol/L	138.55 ± 10.85	140.36 ± 7.60	140.04 ± 6.76	0.008
K, mmol/L	3.84 (3.54, 4.21)	3.84 (3.60, 4.10)	3.84 (3.59, 4.10)	0.257
WBC, ^∗^10^9^/L	19.20 (13.79, 24.19)	8.30 (6.09, 11.67)	10.40 (7.15, 16.54)	< 0.001
NEU, ^∗^10^9^/L	16.75 (10.97, 21.67)	5.91 (3.99, 9.09)	7.71 (4.42, 14.03)	< 0.001
PLT, ^∗^10^9^/L	168.5 (125.5, 216.5)	185.0 (135.5, 217.0)	177.0 (133.0.217.0)	0.146
Hematocrit	0.39 (0.35, 0.43)	0.41 (0.38, 0.45)	0.41 (0.37, 0.44)	< 0.001
BUN, mmol/L	8.20 (6.18, 11.98)	5.84 (4.78, 7.26)	6.30 (5.00, 8.11)	< 0.001
Cr, umol/L	85.50 (67.75, 151.00)	67.00 (56.30, 78.15)	69.10 (58.00, 86.00)	< 0.001
CK, IU/L	1434 (350.00, 4119.25)	132.0 (91.00, 192.50)	173.00 (108.00, 374.00)	< 0.001
LDH, IU/L	789.00 (334.50, 2118.50)	198.00 (170.00, 247.00)	228.00 (185.00, 390.00)	< 0.001
ALT, IU/L	48.50 (26.00, 181.50)	23.00 (16.00, 34.00)	26.00 (18.00, 44.00)	< 0.001
AST, IU/L	220.00 (70.50, 741.25)	26.00 (21.00, 33.50)	31.00 (23.00, 76.00)	< 0.001
DB, umol/L	10.65 (6.48, 17.9)	4.20 (2.40, 6.00)	5.10 (3.00, 9.10)	< 0.001
IB, umol/L	32.85 (15.48, 60.93)	9.30 (6.50, 13.40)	11.30 (7.80, 21.70)	< 0.001
TB, umol/L	46.50 (25.30, 84.38)	13.60 (9.80, 18.55)	16.70 (11.70, 33.00)	< 0.001
PT, S	13.40 (12.10, 14.93)	11.60 (11.00, 12.50)	11.90 (11.20, 13.40)	< 0.001
APTT, S	75.50 (47.98, 120.98)	35.90 (30.95, 44.00)	38.20 (32.40, 70.90)	< 0.001
Fibrinogen, g/L	2.88 (2.40, 3.31)	2.60 (2.29, 2.60)	2.62 (2.31, 3.11)	< 0.001
Death				
No	155 (87.1%)	424 (98.8%)	579 (95.4%)	< 0.001
Yes	23 (12.9%)	5 (1.2%)	28 (4.6%)	
Acute liver injury				
No	80 (44.9%)	411 (95.8%)	491 (80.9%)	< 0.001
Yes	98 (55.1%)	18 (4.2%)	116 (19.1%)	
Acute kidney injury				
No	79 (44.4%)	365 (85.1%)	444 (73.1%)	< 0.001
Yes	99 (55.6%)	64 (14.9%)	116 (19.1%)	
MODS				
No	62 (34.8%)	411 (95.8%)	473 (77.9%)	< 0.001
Yes	116 (65.2%)	18 (4.2%)	134 (22.1%)	
Toxic myocarditis				
No	75 (42.1%)	409 (95.3%)	484 (79.7%)	< 0.001
Yes	103 (57.9%)	20 (4.7%)	123 (20.3%)	
Mechanical ventilation				
No	155 (87.1%)	422 (98.4%)	577 (95.1%)	< 0.001
Yes	23 (12.9%)	7 (1.6%)	30 (4.9%)	
Cardiac arrest in the hospital				
No	170 (98.5%)	428 (99.8%)	598 (98.5%)	< 0.001
Yes	8 (1.5%)	1 (0.2%)	9 (1.5%)	
Hemopurification				
No	68 (38.2%)	401 (93.5%)	469 (77.3%)	< 0.001
Yes	110 (61.8%)	28 (6.5%)	138 (22.7%)	
Time from stings to admission, h	3 (1, 6)	6 (3, 16)	4 (2, 8)	< 0.001

*Note:* APTT: activated partial prothrombin time; ALT: alanine aminotransferase; AST: aspartate aminotransferase; NEU: neutrophil count in peripheral blood; DBP: diastolic pressure; Cr: creatinine; DBil: direct bilirubin; WBC: white blood cell count; PLT: platelet count; LDH: lactate dehydrogenase.

Abbreviations: BUN, blood urea nitrogen; CK, creatine kinase; HR, heart rate; IB, indirect bilirubin; MODS, multiple organ dysfunction syndrome; PT, prothrombin time; SBP, systolic blood pressure; TB, total bilirubin.

^∗^WBC (× 10^9^/L).

### 3.2. Identification of Independent Risk Predictors

**FIGURE 2:**
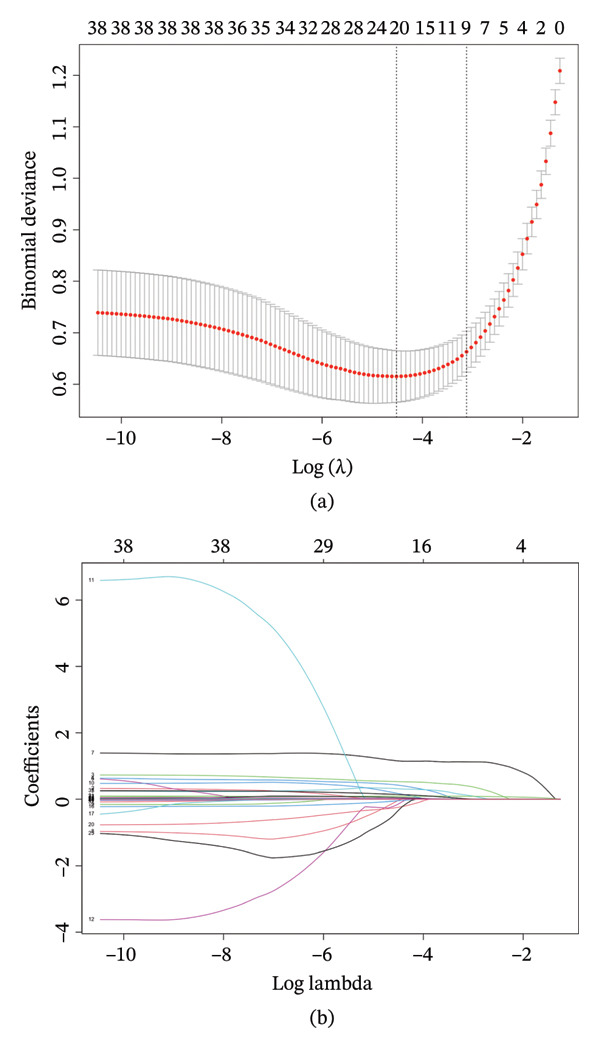
Demographic and clinical features are selected using the LASSO logistic regression model. (A) Optimal lambda parameters for the LASSO model were selected using tenfold cross‐validation and minimum criteria. The binomial deviance curve was plotted against the logarithm of lambda. Dotted vertical lines were placed at the optimal values determined by the minimum criterion and the 1 standard error of the minimum criteria (1‐SE criteria). (B) Coefficient profiles of the nine predictors using LASSO. A plot of the coefficient profile was generated in relation to the logarithmic sequence of lambda. A vertical line was placed at the specified value. The final multivariable logistic regression model was specified as follows: logit(*p*) = −8.559 + 0.030 × age + 0.568 × wasp + 0.486 × (number of stings 15–30) + 0.705 × (number of stings > 30) + 0.877 × tea‐colored urine + 0.093 × WBC + 0.001 × LDH + 0.014 × TB + 0.013 × APTT + 0.263 × month of injury, where *p* represents the predicted probability of rhabdomyolysis.

Based on demographic characteristics, vital signs at presentation, clinical manifestations, and laboratory test results, a total of 9 potential predictive indicators were identified through LASSO analysis(Figure 2A,B). At the optimal lambda value of 0.05 (within 1 standard error from the minimum criterion), 9 variables with nonzero coefficients were identified as potential prediction indicators: age, sting species, number of stings, tea‐colored urine, WBC, LDH, TBIL, APTT, and month of injury. The results of the multivariable logistic regression analysis are presented in Table 2.

**TABLE 2:** Logistic regression was employed to evaluate each variable’s predictive capacity for the risk of wasp venom–induced RM.

	*β*	Prediction model	
		Odds ratio (95% CI)	*p* value
(Intercept)	−8.559	0.000 (0.000–0.002)	0.000
Age	0.030	1.030 (1.011–1.051)	0.003
Wasp	0.568	1.765 (0.874–3.666)	0.118
Number of stings			
15–30 times	0.486	1.626 (0.720–3.525)	0.228
> 30 times	0.705	2.024 (0.916–4.465)	0.080
Tea‐colored urine	0.877	2.403 (0.944–6.207)	0.067
WBC	0.093	1.098 (1.043–1.158)	0.000
LDH	0.001	1.001 (1.000–1.001)	0.046
TB	0.014	1.014 (1.001–1.030)	0.052
APTT	0.013	1.013 (1.006–1.020)	0.001
Month of injury	0.263	1.301 (1.049–1.630)	1.19

### 3.3. Constructing and Validating a Prediction Nomogram Model

**FIGURE 3:**
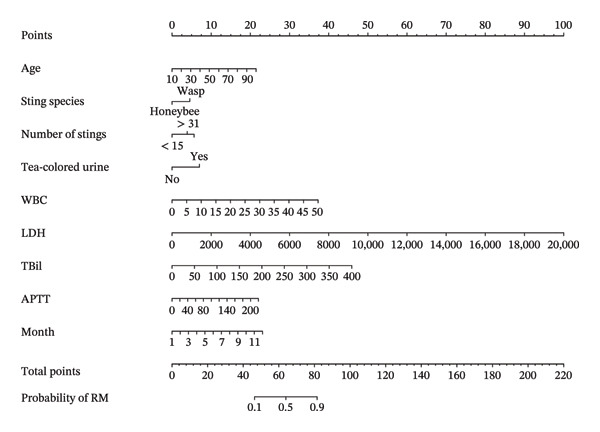
Nomogram to predict the probability of RM in patients with wasp sting. Based on the nomogram points for the number of wasp stings, the initial data line allows for the calculation of age, sting species, number of stings, tea‐colored urine, white blood cell count, LDH, total bilirubin, APTT, and the month of injury. The total score is calculated by summing the individual points for all predictors and is displayed on the “total points” line. The corresponding predicted probability of RM can then be read from the bottom scale.

The 9 selected predictors were used for constructing a predictive nomogram to estimate the risk of RM in the study cohort. The nomogram includes age, sting species, number of stings, tea‐colored urine, WBC count, LDH, TBIL, APTT, and month of injury. A score was assigned to each predictor, and the total score was calculated by summing these individual scores to generate the predicted probability of RM (Figure 3).

**FIGURE 4:**
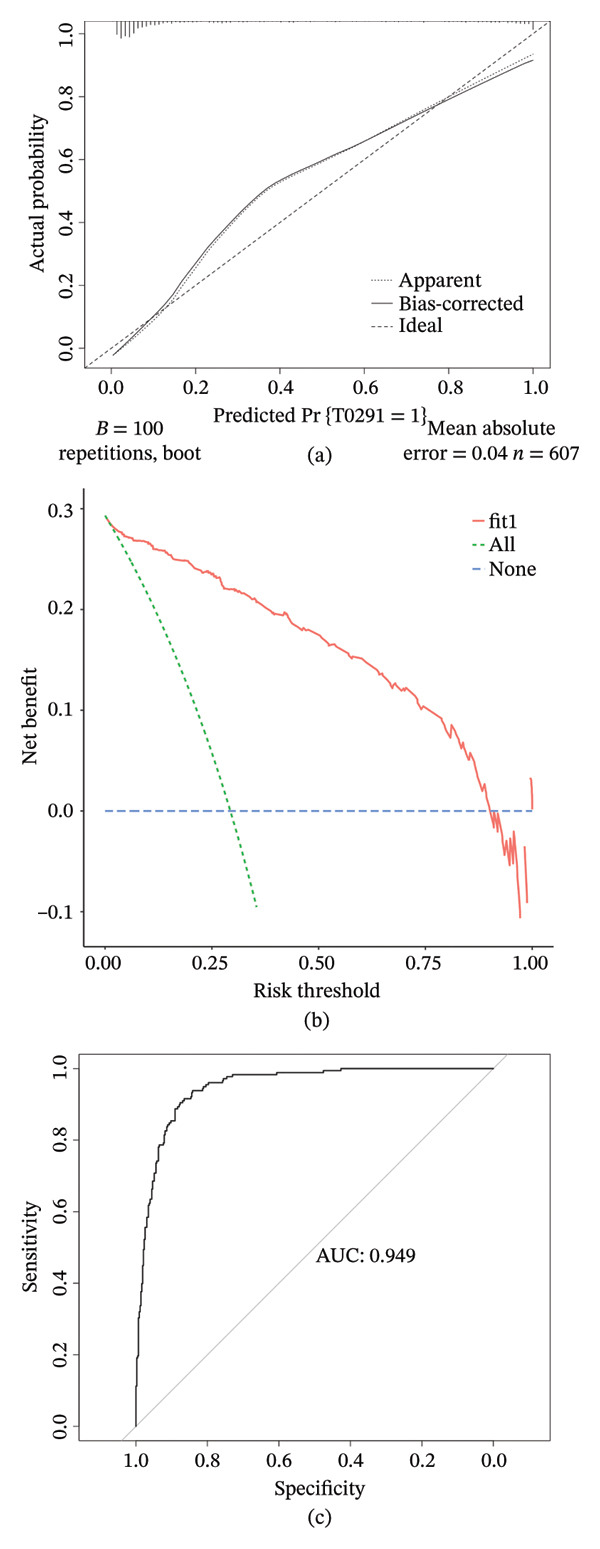
(A) The calibration curves of the nomogram used to predict the probability of RM following a wasp sting. The *x*‐axis represents predicted probability, and the *y*‐axis represents the observed probability. The diagonal dotted line represents the ideal nomogram, where actual and predicted probabilities match. The solid line represents the actual nomogram, while closer alignment with the dotted line signifies improved calibration. (B) Decision curves for the nomogram predicting the probability of RM following a wasp sting. The *x*‐axis denotes threshold probabilities, while the *y*‐axis indicates the net benefit, determined by the sum of true positives minus false positives. The red line represents the predicted column chart. The hypothesis that all patients demonstrate RM is represented by green. The hypothesis that no patients display RM is indicated by the color blue. (C) Receiver operating characteristic (ROC) curve of the nomogram for predicting rhabdomyolysis (RM) in patients with wasp stings.

Model performance was evaluated in terms of discrimination, calibration, and clinical utility. The nomogram’s calibration plots demonstrated optimal agreement between predicted wasp‐induced RM (Figure 4A). DCA assessed the clinical applicability of our prediction nomogram. The nomogram showed good calibration, with the calibration plot demonstrating close agreement between predicted and observed probabilities of RM (Figure 4A). DCA suggested the potential clinical utility of the nomogram, with net benefit observed across threshold probabilities ranging from 0.03 to 0.90 (Figure 4B). The nomogram also showed excellent discrimination, with a concordance index (C‐index) of 0.948 (95% CI, 0.9333–0.968) and an AUC of 0.949 (95% CI, 0.9319–0.9655), indicating strong predictive performance for RM occurrence (Figure 4C). The threshold, sensitivity, specificity, positive predictive value (PPV), and negative predictive value (NPV) were 0.25, 0.90, 0.88, 0.75, and 0.95, respectively.

## 4. Discussion

It is estimated that up to 94.5% of adults experience at least one wasp sting during their lifetime [14]. Although most stings result only in localized reactions, severe or multiple stings may lead to systemic toxic complications, such as anaphylactic shock, RM, multiple organ dysfunction, AKI, hepatic dysfunction, coagulopathy, or death [15–23]. Among these complications, RM is of particular importance because of its close association with AKI and multiorgan dysfunction. In this multicenter retrospective study, we developed a multifactorial predictive model to assess the risk of RM following wasp stings. The model, utilizing nine clinical variables—age, sting species, sting site count, tea‐colored urine, WBC count, LDH, TBIL, APTT, and month of injury—showed excellent discrimination (AUC = 0.949). The study showed that RM was associated with an increased risk of AKI, acute liver injury, MODS, mortality, and greater reliance on mechanical ventilation and hemodialysis. These findings highlight the crucial need for early detection of high‐risk patients. These variables are readily available in routine clinical practice and have good clinical interpretability. To better understand the biological and clinical plausibility of the model, each of the included predictors can be interpreted in light of existing evidence and pathophysiological mechanisms. RM is a syndrome resulting from skeletal muscle damage. It is characterized by the extensive release of cellular contents into the bloodstream, which triggers a series of systemic pathological alterations. The syndrome’s defining features are muscle pain, weakness, and myoglobinuria [8]. With advancing age, both the number and functional capacity of skeletal muscle satellite cells progressively decline, markedly reducing their ability to regenerate following injury [24, 25]. This age‐related decline in regenerative potential renders elderly individuals more susceptible to RM when exposed to external insults such as bee venom. Numerous clinical studies indicate a strong correlation between age and heightened risk of AKI, MODS, and mortality after wasp stings, emphasizing the importance of careful evaluation and management of elderly patients in such cases [7, 15, 26–28]. The prediction model showed that sting species was associated with an increased risk of RM, which may be explained by differences in venom composition and concentration among different bee species. Honeybee and wasp venoms contain distinct bioactive components, which may contribute to variation in systemic toxic effects after envenomation [29–31]. However, because sting species was classified based on patient or family report together with clinical judgment rather than formal entomological identification, some degree of species misclassification may have occurred, potentially affecting the performance and reproducibility of the model. RM and hemolytic reactions following stings are systemic toxic responses directly caused by the venom’s toxic effects. These reactions, also known as venom volume–dependent reactions, do not involve immune mechanisms [29]. Systemic toxic reactions are predominantly attributed to multiple bee stings. However, a small number of case reports document severe systemic toxic reactions arising from a single sting. These reactions may cause organ dysfunction and, in severe instances, escalate to MODS, which can be fatal [32]. In practical clinical work, it is often impossible to calculate the exact amount of venom administered. The toxic impact of wasp venom is generally indicated by the number of stings, as each sting site represents the toxin load [15]. A greater number of stings is linked to an elevated risk of RM. This study showed that sustaining ≥ 30 stings was an important predictive factor for RM. In clinical practice, the number of stings is often used as a surrogate indicator of venom burden, and our findings support the clinical relevance of this threshold. Although the bee sting severity grading criteria applied in this study differ from those used in some previous investigations [3, 15, 33], this approach is reasonable within the present context. There is no universally accepted definition, but our analysis shows a notable rise in RM cases associated with stings exceeding this threshold. We acknowledge that such variations in classification may affect the comparability of results across studies. Future efforts should therefore aim to establish a standardized definition framework for multiple bee stings based on larger sample sizes and multicenter studies, which would enhance both research consistency and clinical applicability.

Tea‐colored urine is a characteristic symptom of RM [34], reflecting the excretion of myoglobin released from damaged muscle cells in the urine. It is an important early indicator of muscle injury. Following wasp stings, tea‐colored urine may appear before laboratory markers such as creatine kinase, demonstrating its high clinical sensitivity.

This study found that patients with wasp sting–induced RM had significantly higher WBC counts. Chemokines in wasp venom attract polymorphonuclear leukocytes, initiating inflammation and inducing cell death [35]. Yuan et al. reported that elevated WBC counts have been linked to AKI after wasp stings [7]. Consistent with this, our study found that patients with RM had a significantly higher incidence of AKI than those without RM (55.6% vs 14.9%). Xie et al. [3] reported that 79.5% of patients with RM from hornet stings developed AKI, exceeding our observed rate of 55.6%. LDH is a widely distributed marker of cellular injury found in cardiac and skeletal muscles, as well as hepatocytes, with its serum activity indicating the level of cell damage. Evidence indicates that LDH is crucial in the progression of inflammation and AKI, also serving as a prognostic marker for RM‐induced AKI [36].

Wasp stings show a distinct seasonal pattern, with summer and autumn representing peak periods of bee activity and, consequently, high‐risk months for stings and related severe complications [37]. Our study found that patients with RM had significantly higher rates of AKI, acute liver injury, MODS, and in‐hospital mortality than those without RM. The underlying mechanisms may include renal vasoconstriction, intraluminal cast formation, and direct injury to renal tubular cells [38], as well as bee venom–induced hepatocellular damage and multiorgan dysfunction resulting from systemic inflammatory responses and metabolic disturbances.

Moreover, patients with RM often experience rapid disease progression and demonstrate a greater dependence on mechanical ventilation and hemodialysis support [39]. The associated treatment costs and complexity increase accordingly. Therefore, identifying individuals at high risk of RM following wasp stings is essential for the early initiation of fluid resuscitation, urine alkalinization, renal function monitoring, and intensive care management. Such early interventions can effectively improve patient outcomes and reduce mortality. The present nomogram is intended to serve as an adjunctive tool for early risk stratification in adult patients presenting to the emergency department after wasp stings. By estimating the probability of RM at an early stage, the model may help clinicians identify patients who may benefit from hospital admission, closer monitoring, serial laboratory testing, renal function surveillance, and timely aggressive fluid therapy. In contrast, patients with a lower predicted risk and stable overall clinical condition may be managed with less intensive observation and follow‐up. This study advances previous research on wasp sting–related RM by developing a predictive model using extensive multicenter data, enhanced with a nomogram for visualization, thereby ensuring scientific rigor and practical applicability. The model uses readily available clinical variables and may be useful in emergency departments and related acute care settings. However, further external validation is required before broader clinical implementation. By helping identify high‐risk patients at an early stage, the model may support individualized management, more efficient resource allocation, and improved clinical decision‐making.

## 5. Conclusion

In summary, the predictive model for RM following wasp stings developed in this study identified nine clinically meaningful predictors and showed that RM was associated with an increased risk of severe complications and mortality. The model includes age, bee species, number of sting site, tea‐colored urine, WBC count, LDH, TBIL, APTT, and month of injury. This model may serve as an important tool for the early clinical identification of patients at high risk of RM after wasp stings, providing theoretical support and practical guidance for precise management and individualized intervention.

### 5.1. Limitation

Despite the multicenter design and an adequate sample size, several limitations should be acknowledged. First, as a retrospective analysis, some clinical data were incomplete, which may have affected the comprehensiveness of variable selection. Second, myoglobin has been identified as a prognostic biomarker for RM [34]. However, due to incomplete myoglobin data, it was not incorporated into the model. Future studies should supplement and validate this variable. Third, this study included only adult patients because pediatric wasp sting cases were insufficient across the five participating centers. Therefore, the current nomogram is intended for adult emergency department patients only, and its generalizability to pediatric populations is limited. Future studies in children are needed. Fourth, the current model has not yet been validated in an external cohort, and its generalizability requires further evaluation. Prospective multicenter validation and the development of dynamic predictive modeling are warranted to enhance the model’s accuracy and applicability.

## Author Contributions

Xiaoyan Xian: data curation (equal); formal analysis (equal); investigation (equal); project administration (equal); writing–original draft (lead).

Guoqiang Chen: data curation (equal); writing–review and editing (equal).

Jianping Hu: data curation (equal); writing–review and editing (equal).

Yuanjun Zhang: data curation (equal); writing–review and editing (equal).

Mengqin Li: data curation (equal); writing–review and editing (equal).

Jing Chen: data curation (equal); writing–review and editing (equal).

Jiao Li: data curation (equal); writing–review and editing (equal).

Shuyun Xu: project administration (lead); writing–review and editing (lead).

## Funding

This work was funded by Chengdu High‐Tech Zone Medical Research Project (No. 2025003).

## Disclosure

All authors approved of publication.

## Ethics Statement

The study complied with the Declaration of Helsinki principles and obtained ethical approval from the Ethics Committee of West China Hospital, Sichuan University (No. 2014156). Informed consent was waived due to the de‐identification of all patient data.

## Conflicts of Interest

The authors declare no conflicts of interest.

## Data Availability

The dataset supporting the conclusions of this article is available from the corresponding authors upon request.
